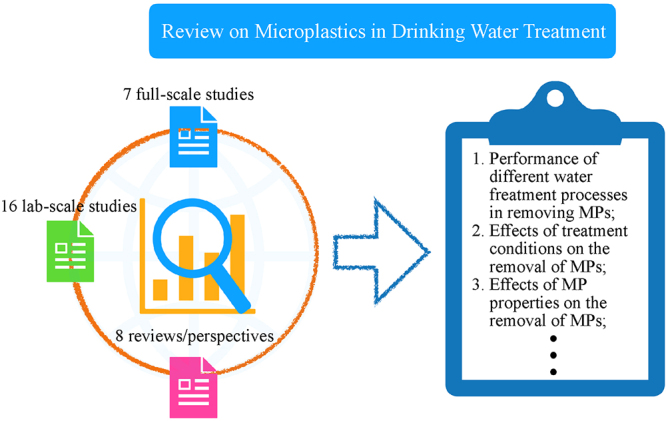# What have we known so far about microplastics in drinking water treatment? A timely review

**DOI:** 10.1007/s11783-021-1492-5

**Published:** 2021-10-15

**Authors:** Jinkai Xue, Seyed Hesam-Aldin Samaei, Jianfei Chen, Ariana Doucet, Kelvin Tsun Wai Ng

**Affiliations:** Environmental Systems Engineering, Faculty of Engineering & Applied Science, University of Regina, 3737 Wascana Parkway, Regina, SK S4S 0A2 Canada

**Keywords:** Microplastics, Drinking water treatment, Coagulation, Flocculation, Membrane, Filtration

## Abstract

Microplastics (MPs) have been widely detected in drinking water sources and tap water, raising the concern of the effectiveness of drinking water treatment plants (DWTPs) in protecting the public from exposure to MPs through drinking water. We collected and analyzed the available research articles up to August 2021 on MPs in drinking water treatment (DWT), including laboratory- and full-scale studies. This article summarizes the major MP compositions (materials, sizes, shapes, and concentrations) in drinking water sources, and critically reviews the removal efficiency and impacts of MPs in various drinking water treatment processes. The discussed drinking water treatment processes include coagulation-flocculation (CF), membrane filtration, sand filtration, and granular activated carbon (GAC) filtration. Current DWT processes that are purposed for particle removal are generally effective in reducing MPs in water. Various influential factors to MP removal are discussed, such as coagulant type and dose, MP material, shape and size, and water quality. It is anticipated that better MP removal can be achieved by optimizing the treatment conditions. Moreover, the article framed the major challenges and future research directions on MPs and nanoplastics (NPs) in DWT.

## 1 Introduction

Microplastics (MPs) are an urgent issue due to their ubiquity and potential adverse impacts on the environment and human health (Wright and Kelly, 2017; Asmonaite et al., 2018; Zhang et al., 2020). MPs are defined as plastic beads, fibers, and fragments that are within a size range of < 5 mm (Pham et al., 2021). They enter the environment through various pathways, such as municipal and industrial wastewater, surface runoff, and continuous breakdown of larger plastics in the environment (Ziajahromi et al., 2017; Duan et al., 2021; Sun et al., 2021). Resultantly, MPs are present in almost all water bodies. Despite a lack of knowledge, preliminary studies have shown that MPs can lead to health problems of different organisms (Sussarellu et al., 2016; World Health Organization, 2019; Prata et al., 2020; Zhang et al., 2021a). In addition, the hydrophobicity of MPs and their complex surface characteristics make them potential vectors for other pollutants, such as micropollutants, heavy metals, and pathogens, further worsening their environmental and health impacts (Frère

✉ Corresponding author
E-mail: jinkai.xue@uregina.ca

Special Issue—Frontier Progresses from Chinese-American Professors of Environmental Engineering and Science (Responsible Editors: Xing Xie, Jinkai Xue & Hongliang Zhang)

et al., 2018; World Health Organization, 2019; Pham et al., 2021). Therefore, it is imperative to evaluate how MPs are prevented from threatening humans through drinking water. Yet, research on MPs in drinking water treatment (DWT) remains insufficient.

Although MPs are one of the hottest research topics in environmental science and engineering in the recent decade (Zhu et al., 2021), the number of studies on MPs in DWT is limited. We found only 31 published studies on MPs in DWT within the period of January 2018 to August 2021, including 23 research articles and eight review or perspective articles (Fig. 1). As is indicated in Fig. 1A, these studies on MPs in DWT were conducted by researchers (first or correspondent authors) from 11 countries, including China, Canada, Germany, UK, and the Czech Republic. In Fig. 1B, the total number of studies on MPs in DWT substantially rose over the past three years. Among these studies, coagulation-flocculation (CF) is the most frequently examined DWT process for MP removal, followed by membrane and media filtration. These treatment processes are arguably the most widely used DWT technologies (Fig. 1C). The scarce quantity of studies on MPs in DWT is partially attributed to the difficulty in detecting and quantifying the relatively small sizes (low micrometer-scale, e.g., < 20 μm (Mintenig et al., 2019)) and low concentrations of MPs (e.g., < 20 MPs/L (Johnson et al., 2020)) in DWT. The small MPs in DWT requires specialized detection instruments, such as Fourier transform infrared microscopy coupled with a focal plane array (FPA-micro FTIR) (Prata et al., 2019) and pyrolysis-gas chromatography-mass spectrometry (pyrolysis-GC-MS) (Gomiero et al., 2021). The low concentrations

**Fig. 1** Bibliometrics on MPs in DWT over the period of January 2018 – August 2021: (A) Numbers of research articles according to the first and correspondent authors' countries, (B) Annual numbers of published studies on full-scale DWTPs, laboratory-scale tests, and literature reviews or perspectives, as well as the totals (up to August 2021), (C) Comparison of the numbers of research papers that cover CF/CFS, membrane, and media filtration.

warrant large volumes of samples for reliable measurements (Uhl and Dadkhah, 2018; Lv et al., 2019). To better protect public health, more studies on MPs in DWT are needed.

Despite the small number of research articles on this topic to date, there are already eight review or perspective papers dealing with MPs in DWT in the past three years (Fig. 1B). Due to the limited pertinent studies, these previous reviews had to derive discussions and conclusions extensively from studies that were focused on MPs in surface water bodies and wastewater treatment (Enfrin et al., 2019; Novotna et al., 2019; Barchiesi et al., 2020; Shen et al., 2020; Cheng et al., 2021; Ding et al., 2021; Rodríguez-Narvaez et al., 2021; Xu et al., 2021). These preliminary yet valuable reviews have indicated that 1) DWTPs are critical in protecting the public from MP exposure; 2) conventional DWT processes are potential for MP removal; and 3) more investigation is needed in the efficiency of different DWT processes in removing MPs. To date, in-depth review discussions on the effects of treatment conditions (such as coagulant type and dose) and the properties of MPs on the removal of MPs remain rare. Plus, some of the latest studies on MPs in DWT are not yet reviewed. We feel it is necessary and opportune to conduct a critical review over the 23 studies on MPs in DWT.

Therefore, the objectives of this review are: 1) to summarize the MP compositions in DWT in terms of material, size, and shape; 2) to summarize the MP removal efficiencies by a number of DWT processes, such as CF, membrane filtration, and media filtration; 3) to explore the influential factors to MP removal in DWT; and 4) to identify the challenges and research opportunities regarding MPs in DWT.

## 2 Major MPs and their removal at DWTPs

### 2.1 Compositions of MPs in raw waters of DWTPs

MPs are highly diverse in source waters to DWTPs in terms of material, size, shape, concentrations, etc. Denser materials (e.g., polyester (PEST) and nylon fibers) that are negatively buoyant in water may remain suspended in turbulent water bodies, such as a river, and settle out to the sediments in quiescent lakes (Baldwin et al., 2016; Lenaker et al., 2020). In contrast, lighter polymers, such as PS, PE, and PP pellets/fragments/films, may stay suspended in lakes until they become denser when associated with other matters, such as biofilms and minerals (Baldwin et al., 2016). Other than the intrinsic physical/chemical properties of polymers, the presence of MPs in certain locations in water bodies is also determined by turbulence, formation of biofilms, and adsorption of inorganics (Anderson et al., 2016). In addition, particle shapes can be important in determining the distribution of MPs in the water column (World Health Organization, 2019). This section will summarize the diversity of MPs in DWTP source waters in the published studies on full-scale DWTPs. The inconsistency in sampling and measurement methodologies among studies has made quantitative comparisons difficult, but some qualitative conclusions can be established.

The compositions of MPs vary temporally, spatially, and geographically, yet certain MP materials (polymer types) are repeatedly detected in raw waters of DWTPs across the world. Globally, the most commonly detected MPs in surface water are polyethylene (PE), polypropylene (PP), polystyrene (PS) and poly(ethylene terephthalate) (PET), polyvinyl chloride (PVC), PEST, nylon, and cellulose acetate (CA) (Zhang et al., 2015; Andrady, 2017; Hendrickson et al., 2018; Wang et al., 2018; Shen et al., 2021), which is in accordance to the order of magnitude of production of these materials. Table 1 summarizes studies on MP removal at full-scale DWTPs (Pivokonsky et al., 2018; Mintenig et al., 2019; Johnson et al., 2020; Pivokonský et al., 2020; Wang et al., 2020a; Dalmau-Soler et al., 2021; Sarkar et al., 2021). To better show the frequency (rather than abundance) of each material detected in the studies, a word-cloud image (Fig. 2) has been generated with larger font sizes indicating higher detection frequencies. Based on the seven studies on full-scale DWTPs, PE, PP, PET, PVC, and PS are the most widely detected MPs (with a size over 1 μm) in DWTP source waters (Table 1). These materials are commonly used all over the world: PE for bags, packaging, and houseware; PP for packaging and pipes; PET for beverage bottles; PVC for construction and pipes; and PS for packaging, toys, appliances, etc. (Shah and Jan, 2014; Pivokonsky et al., 2018). PET has been most frequently reported as the prevailing polymer type (generally 55–68% of total MPs) as compared with other major polymers (Pivokonsky et al., 2018; Wang et al., 2020a; Sarkar et al., 2021). PE and PP are usually the second and third predominant polymers, respectively, in DWTP raw waters (Pivokonsky et al., 2018; Wang et al., 2020a; Sarkar et al., 2021). In contrast, CA was the predominant MP material in the raw waters at the two Czech DWTPs in Pivokonský et al. (2020), and PE was predominant in the raw waters at most of the DWTPs in Johnson et al. (2020). Moreover, due to the global use of disposable masks during the COVID-19 pandemic, substantial increases in concentrations of these polymers consisting in masks (such as PEST, PP, and PS) are anticipated in fresh water sources in the following years (Xu et al., 2021).

MPs are also diverse in size with the smaller ones generally being much more abundant and prevalent (Andrady, 2017). Smaller MPs, especially when close to or within the colloidal size range, tend to be more stable in the water column and hard to detect using the current methodologies (Lv et al., 2019). This poses a bigger challenge to researchers and water/wastewater engineers. To date, the studies on MPs at DWTPs have been focused

**Table 1** Summary of studies on full-scale DWTPs

| Authors | Country | Water source | Microplastic composition | Treatment processes | Overall performance remark |
|---|---|---|---|---|---|
| Mintenig et al. (2019) | Germany | Groundwater | Materials: PEST, PVC, PE, PA/nylon, and epoxy resin; Size range: 50–150 μm; Concentration: 0–7 MPs/$m^3$ | Aeration-serial filtration | MPs in treated water: 0–0.7 MPs/$m^3$ |
| Sarkar et al. (2021) | India | Ganga River water | Materials: PE, PP, PET, and PS; Dominant sizes: ⩾25 μm; Shapes: fibers, 52%–59%; films/fragments, 41%–48%; Concentration: 17.88 MPs/L | CF-pulse clarifier-sand filtration | CF-pulse clarifier: 60.9% removal; CF-pulse clarifier-sand filtration: 84.6% removal; MPs in treated water: 2.75 MPs/L |
| Pivokonský et al. (2020) | The Czech Republic | Uhlava River water | Materials: CA, PET, PVC, PE, PP, EVA, PBA, PTT; Size range: 1–100 μm and ⩾100 μm; Shapes: fibers, fragments, and spheres; Concentration: ~23 MPs/L | CF-sand filtration | ~40% removal of MPs 1–100 μm; MPs in treated water: 14 MPs/L |
| | | | Materials: CA, PET, PVC, PE, PP, EVA, PS, PA6, PEO + PEG, VC/VAC, PTT, PTFE; Size range: 1–100 μm and ⩾100 μm; Shapes: fibers, fragments, and spheres; Concentration: ~1300 MPs/L | CFS-Mn oxidation-sand filtration-ozonation-GAC filtration-UV absorption | Up to 88% removal of MPs 1–100 μm; MPs in treated water: 160 MPs/L |
| Pivokonsky et al. (2018) | The Czech Republic | Reservoir water | Major materials: PET, PP, PS, PVC Size range: 1–100 μm and ⩾100 μm; Shapes: fragments, spherical, and fibers Concentration: ~1473 MPs/L | CF-sand filtration | ~70% removal of MPs 1–100 μm; MPs in treated water: ~443 MPs/L |
| | | Reservoir water | Major materials: PET, PP, PVC Size range: 1–100 μm and ⩾100 μm; Shapes: fragments, spherical, and fibers Concentration: ~1812 MPs/L | CFS-sand and granular activated carbon filtration | ~81% removal of MPs 1–100 μm; MPs in treated water: ~338 MPs/L |
| | | River water | Major materials: PBA, PE, PET, PMMA, PP, PS, PTT Size range: 1–100 μm and ⩾100 μm; Shapes: fragments, spherical, and fibers Concentration: ~3605 MPs/L | CF-flotation-sand filtration-granular activated carbon filtration | ~83% removal of MPs 1–100 μm; MPs in treated water: ~628 MPs/L |
| Johnson et al. (2020) | UK | River water | Material: PE; Size range: ⩾25 μm; Concentrations: 0–4.4 MPs/L | GAC-membrane-UV/$H_2O_2$-GAC-disinfection | MPs in treated water: 0 MPs/L* |
| | | River water | PE: ⩾25 μm, 0–15 MPs/L; PP: ⩾25 μm, 0–3.4 MPs/L | HBC-RGF-GAC-disinfection | MPs in treated water: 0 MPs/L |
| | | River water | PE: ⩾25 μm, 0–1.8 MPs/L; | Disinfection-pH balancing-static mixer-clarifier with $FeCl_3$-polyelectrolyete-coagulation-RGF-GAC-microscreen | MPs in treated water: 0 MPs/L |
| | | River water | PE: ⩾25 μm, 0–113 MPs/L; PET: ⩾25 μm, 0–20 MPs/L; PP: ⩾25 μm, 0–1.3 MPs/L | DAF or HBC-RGF-GAC-disinfection | MPs in treated water: 0 MPs/L |
| | | River water | PE: ⩾25 μm, 0–0.2 MPs/L | Reservoir with SSF-RGF-ozone-SSF-disinfection | MPs in treated water: PS 0.0016 MP/L |
| | | Groundwater | – | Disinfection | PS and ABS were detected, totally 0.0028 MP/L |
| | | Groundwater | – | Aeration and pressure-filtration-disinfection | MPs in treated water: 0 MPs/L |
| | | Reservoir water | PP: ⩾25 μm, 0–0.023 MPs/L | Alum coagulation-RGF-disinfection-pH balancing-UV | MPs in treated water: 0 MPs/L |

(*Continued*)

| Authors | Country | Water source | Microplastic composition | Treatment processes | Overall performance remark |
|---|---|---|---|---|---|
| Wang et al. (2020a) | China | Yangtze River water | Materials: PET, PE, PP, PAM<br>Size range: 1–100 μm and ⩾100 μm<br>Shapes: fibers, spheres, and fragments<br>Concentration: ~6614 MPs/L | CFS-sand filtration-ozonation-GAC filtration | PET: ~87%;<br>PE: ~89.5%;<br>PP: ~85.0%;<br>PAM: −114.1%;<br>MPs in treated water: ~930 MPs/L |
| Dalmau-Soler et al. (2021) | Spain | Llobregat river basin river and ground-water | Materials: PP, PEST, PS, PAN, ABS, PE<br>Size: 20 μm–5mm<br>Shape: fragments, fibers<br>Concentration: ~1 MPs/L | [CFS-sand filtration] + $\frac{\text{Line1: ozonation} - \text{GAC filtration}}{\text{Line 2: UF} - \text{RO}}$ | Overall removal: ~93%;<br>MPs in treated water: ~0.07 MPs/L |

*>LOQ (limit of quantification).

on MPs with a size range that is larger than 1 μm due to the limitations of the current detection methodologies. Despite the different size ranges measured in the previous studies, it is widely documented that smaller MPs tend to dominate in DWTP raw waters (Pivokonsky et al., 2018; Pivokonský et al., 2020; Wang et al., 2020a; Sarkar et al., 2021). According to Wang et al. (2020a), 54.6%–58.0% of the detected MPs had a size range of 1–5 μm, while 20.0%–27.6% were within 5–10 μm. Pivokonsky et al. (2018) reported that MPs smaller than 10 μm prevailed in the raw waters at all the studied DWTPs. It is therefore conceivable that MPs smaller than 1 μm, termed nanoplastics (NPs), can be much more abundant in DWTP raw waters. Effective detection methods for NPs and more investigations are thus needed to evaluate the risks and removal of small MPs (e.g., < 10 μm) and NPs in DWTPs.

**Fig. 2** Word-cloud of the major detected MP materials in raw waters to DWTPs. (Font sizes are determined by the frequencies of detection of the materials in all the studies. For example, PE was reported in all the seven studies on full-scale DWTPs, so PE's frequency is 7. Colors are only for aesthetics. The image was generated using WordItOut at https://worditout.com).

The most widely reported MP shapes in DWTP raw waters are fragments/films, fibers, and spheres (Table 1). Fragmented MPs are mostly secondary MPs that are generated from the weathering processes of larger plastics, thus there is high diversity of fragmental MPs in terms of source and chemical composition (Pivokonský et al., 2020; Wang et al., 2020a; Sarkar et al., 2021). Fibers are mainly from wastewater (both treated and untreated) discharged into the natural water environment (Cai et al., 2020b). They can be further traced back to the laundry of synthetic garments. According to De Falco et al. (2019), a single wash per kilogram of fabric can release 640000 to 1500000 fibers. As for spherical MPs, they largely originate from personal care and cosmetic products (Wang et al., 2020a; Wang et al., 2020b). Even though some countries such as Canada have banned the use of plastic microbeads in personal care products, spherical MPs will remain in the environment for a very long time due to the inertness of the polymers. Among all the shapes, fragments have been more frequently documented as the predominant MP shape in DWTP raw waters. Pivokonsky et al. (2018) found that fragments took up 71%–76% of the total MPs in raw waters at two DWTPs, followed by fibers and spheres. At the Milence DWTP in Pivokonský et al. (2020), 80% of the total detected MPs in raw water were fragments and 20% were fibers. In the other DWTP receiving source water from the same river, fragments accounted for 87%–92% of the total MPs in the raw water (Pivokonský et al., 2020). In contrast, spherical MPs were not detected in the raw waters of the two DWTPs in Pivokonský et al. (2020). However, in some cases, fibers were more abundant than fragments in DWTP raw waters. One of the three DWTPs in Pivokonsky et al. (2018) showed that 37%–61% of the total MPs were fibers. In Sarkar et al. (2021) and Wang et al. (2020a), fibers represented 52%–59% and 54%–74% of the total MPs in raw water, respectively. Generally, fibers are made from denser polymers, such as CA (1.3 g/cm$^3$), PEST (1.38 g/cm$^3$), and nylon (Nylon 6,6 density: 1.15 g/cm$^3$), thus they are heavier than many fragmented and spherical MPs (such as PE, 0.857–0.975 g/cm$^3$) and tend to settle in water bodies. Moreover, according to Pivokonský et al. (2020), the size distributions of MPs differ greatly between shapes. In that study, MP fragments skewed toward the range of 1–5 μm in raw water at both the DWTPs; whereas fibers tended to be larger: mostly ⩾50 μm at the Milence DWTP and ⩾5 μm at the Plzen DWTP (Pivokonský et al., 2020). In addition to the actual shape composition of MPs in the source water body (including the water column and sediment), the shape composition of MPs in sampled raw water is influenced by the location- and time-specific hydrodynamic conditions. Moreover, the inconsistency in defining the shape categories of MPs should be noted. MP fragments in one study can be categorized as MP films or pellets in another. Thus, there is a need to standardize the definitions of MP shapes to ensure the comparability of findings from different studies.

The concentrations of MPs can substantially differ between water sources, different locations of the same water body, or sampling times (Lenaker et al., 2020). Highly different MP concentrations over a range of 0–6614 MPs/L have been reported for different drinking water sources (Fig. 3). In Pivokonsky et al. (2018), the MP concentrations (⩾1 μm) of raw waters from a large water reservoir, a small reservoir, and a river were ~1473, ~1812, and ~3605 MPs/L, respectively. In Pivokonský et al. (2020), two DWTPs sourcing from the same river (Uhlava River, Czechia) had very different MP (⩾1 μm) concentrations (~23 vs. ~1296 MPs/L on average) in their raw waters. Wang et al. (2020a) detected ~6 614 MPs/L (⩾1 μm) in Yangtze River water in China. Johnson et al. (2020) surveyed the MPs (⩾25 μm) in raw waters of eight DWTPs, with each being visited and sampled five times. The authors found variable raw water MP concentrations for each DWTP. For example, the quantifiable MP concentration of the raw water of the DWTP identified as LRS1 was 113 MPs/L for Visit 4 and close to 0 for the other visits.

For MP studies, the biggest challenge is the detection limit of the currently available methodologies. For example, FTIR with proper configurations (such as focal plane array-micro FTIR) is generally able to identify MPs down to 10–20 μm, and micro-Raman spectroscopy can identify MPs with a size as small as 1 μm (Delgado-Gallardo et al., 2021). Although novel methods have been reported for the detection of NPs (1–1000 nm), quantitative methods that are capable of detecting environmental NPs and differentiating polymer types are yet to be developed (Cai et al., 2021). Due to this reason, it remains unknown what plastic particles (e.g., materials) below 1-μm are present in DTWP raw waters. In addition, the sampling, extraction, detection, and categorizing methodologies vary greatly across studies, making the findings less comparable. More effective protocols of sampling and extraction and advanced detection techniques with much stronger resolution capability (down to several nm), such as hyperspectral imaging (HSI) and Raman spectroscopy with Raman tweezers, can be useful for MP detection in water (Delgado-Gallardo et al., 2021; Nigamatzyanova and Fakhrullin, 2021). Moreover, it can be helpful to evaluate the abundance of MPs in water by mass

**Fig. 3** Reported MP concentrations in different source waters to full-scale DWTPs in different countries: a groundwater in Germany (Mintenig et al., 2019), the Ganga River (Sarkar et al., 2021), two locations of the Uhlava River in the Czech Republic (Czechia) (Pivokonský et al., 2020), two reservoirs and a river in Czechia (Pivokonsky et al., 2018), a river in the UK (Johnson et al., 2020), the Yangtze River in China (Wang et al., 2020a), and the Llobregat River and its tributaries in Spain (Dalmau-Soler et al., 2021). It should be noted that this chart is only to showcase how substantial MP concentrations may differ across studies; even for the same water body, the MP concentrations measured at different locations can differ significantly.

**Fig. 4** Representative DWT trains that contain CF treatment: a conventional DWT consisting of CF, sand filtration, and $Cl_2$ disinfection (A); and two advanced DWT trains, which are (B) CFS-sand filtration-ozonation-GAC filtration-UV-$Cl_2$ disinfection and (C) CFS-sand-filtration-membrane filtration- $Cl_2$ disinfection.

concentration. With the aid of novel detection techniques and more consistent methodologies, better understandings of MPs and NPs in DWTP raw waters are expected.

### 2.2 Overall removal of MPs at DWTPs

A variety of processes can be incorporated in DWT, including coagulation-flocculation (CF), sedimentation, membrane filtration, granular media filtration (such as sand filtration and granular activated carbon filtration), ozonation, and UV (Table 1). Fig. 4 presents three representative DWT trains that contain CF and have been examined for MP removal (Pivokonsky et al., 2018; Pivokonský et al., 2020; Wang et al., 2020a; Dalmau-Soler et al., 2021). According to the available studies, existing DWTPs are generally able to remove over 80% of MPs with a size of 1–100 µm (Table 1) (Pivokonsky et al., 2018; Pivokonský et al., 2020; Wang et al., 2020a; Sarkar et al., 2021). Most of the DWTPs in these studies had coagulation-flocculation-sedimentation (CFS) and sand filtration (Table 1 and Fig. 4). In general, more treatment steps are beneficial to the removal of MPs. DWTPs consisting of CF and sand filtration exhibited an overall MP removal of ~40%–~70% (Pivokonsky et al., 2018; Pivokonský et al., 2020). In contrast, DTWPs with more steps, such as CFS-sand filtration-ozonation-GAC filtration, achieved >80% removal of MPs (Pivokonsky et al., 2018; Pivokonský et al., 2020; Wang et al., 2020a). In Sarkar et al. (2021), a DWTP consisting of CF-pulse clarifier-sand filtration removed 84.6% MPs from the raw water containing ~17.88 MPs/L. It should be noted that some treatment processes may actually contribute MPs to the treated water. Specifically, the use of polymer coagulant aid in the CFS process and the polymeric membranes may conceivably result in higher concentrations of MPs in the treated water. Wang et al. (2020a) found the CFS process resulted in a 114%-increase in PAM (commonly used coagulant aid) concentrations in the water. Dalmau-Soler et al. (2021) detected MP materials in the treatment plant, which had not been detected in the raw water, such as PTFE and epoxy resins. The difference in MP removal efficiency at different DWTPs can be related to the different treatment processes. However, the influence of MP compositions and concentrations as well as water characteristics should also be considered. Detailed discussion on the removal of MPs by different treatment processes and the influence of various factors are provided in the following context.

## 3 MP removal by CF-based treatment

CF, often followed by sedimentation (CFS), is the most widely used drinking water treatment method to remove particulate matter and a fraction of natural organic matter (NOM) from water (Crittenden et al., 2012). Coagulation refers to the process of adding a chemical coagulant or coagulants for conditioning (destabilizing) the particulate, colloidal, and dissolved matter for the following flocculation step (Crittenden et al., 2012). It occurs rapidly (e.g., < 10 s). Flocculation involves aggregation of destabilized particles and the participation products of coagulant (s) by extended (e.g., 20–45 min) slow mixing, resulting in larger flocs that can be readily removed by sedimentation or filtration (Crittenden et al., 2012). Out of its wide-spread

**Table 2** MP removal by CF at full-scale DWTPs

| Study | Source water | Treatment step | MP concentration (MPs/L) | MP size range | Overall removal (%) |
|---|---|---|---|---|---|
| Sarkar et al. (2021) | River water | CF-pulse clarifier | 17.88 | $< 100$ μm | 60.9 |
| Pivokonský et al. (2020) | River water | CFS | ~1300 | 1–100 μm | 61.5 |
| Wang et al. (2020a) | River water | CFS | ~6614 | 1 –>100 μm | Overall: 40.4–54.5<br>$> 100$ μm: 100<br>50–100 μm: 100<br>10–50 μm: 68.4 – ~100<br>5–10 μm: 44.9–75.0<br>1–5 μm: 21.5–34.2 |

application in modern DWT, CF's efficiency in removing MPs is worth comprehensive investigation. Out of the 23 research articles on MPs in DWT, 19 articles examined the CF-based process (CF and CFS) (Fig. 1). As is summarized in Table 1, among the 21 DWTPs covered by the seven studies, 10 had CF. Table 2 demonstrates the overall removal of MPs by CF at full-scale DWTPs. Table 3 summarizes the 16 published studies on MP removal in laboratory-scale DWT, in which 13 of them examined the removal of MPs in CF. Despite the different concentrations and compositions of MPs in raw waters, the three full-scale DWTPs in three different countries exhibited comparable removal percentage of MPs (40.4%–61.5%) by CF treatment (Table 2). The MP removal by laboratory-scale CF tests varied more remarkably among studies (Table 3). Nonetheless, it is common to see an MP removal of $> 60\%$ in Table 3. Because of a number of factors that differed among these studies, such as source water characteristics, MP compositions, coagulant type and dose, operating conditions, and sampling and quantification methods, directly comparing the removal percentages among these studies will provide no fair conclusion. However, these preliminary studies offer an opportunity to draw insights into the effects of various factors on the removal of MPs in DWT. In this section, we discuss MP removal by CF (mostly CFS) on the basis of publications examining both full-scale and laboratory-scale studies.

### 3.1 Effects of coagulant and coagulant aid

Some laboratory-scale studies compared MP removal by CFS with different types and doses of coagulant or coagulant aids, allowing us to explore their effects on MP removal.

The coagulants that have been used for MP removal in laboratory- and full-scale drinking water treatment are generally alum, polyaluminum chloride (PACl) (also termed aluminum chloride in different studies), aluminum chlorohydrate (ACH), and ferric chloride (as $FeCl_3 \cdot 6H_2O$) (Table 3). Some coagulants may be more effective than others in removing certain MP materials. Zhou et al. (2021) compared PACl and $FeCl_3$, and found that the former was more effective than the latter in removing PS and PE MPs (e.g., ~78% removal of PS by PACl (90 mg/L) vs. ~64% removal of PS by $FeCl_3$ (90 mg/L); ~30% removal of PE by PACl (90 mg/L) vs. 17% removal of PE by $FeCl_3$ (90 mg/L)). Ma et al. (2019b) examined the removal of PE MPs by coagulation processes using $FeCl_3 \cdot 6H_2O$ or $AlCl_3 \cdot 6H_2O$. They found that $AlCl_3 \cdot 6H_2O$ was more effective in removing the PE MPs than $FeCl_3 \cdot 6H_2O$. In addition, the study showed that the removal of PE MPs reached the maximum at a much lower $FeCl_3 \cdot 6H_2O$ dose than it did with $AlCl_3 \cdot 6H_2O$. It seems that PACl is more effective in removing MPs than $FeCl_3$. Such a difference may result from the variations in the strength of the hydrophobic and electrostatic interactions between the MPs and coagulant flocs, and further investigation is required to unveil the actual mechanisms. Nevertheless, it should be noted that Ma et al. (2019b) and Zhou et al. (2021) used model PE and PS MPs with un-weathered surfaces and fairly large particles sizes (0.5–5 mm) and synthetic water. In contrast, MPs in real DWTP source waters are highly diverse. As real waters are much more complex, it is less likely that a coagulant that is universally more effective for all MPs in all waters exists. Nonetheless, novel coagulants that are more cost-effective and environmentally friendly for MP removal can still be developed. More recently, Peydayesh et al. (2021) introduced a novel bio-material lysozyme amyloid fibrils as the coagulant to remove MPs from various water matrices and documented 98.2% removal of carboxylated PS MPs (500 nm) at a fairly low coagulant dose (12.5 mg/L).

Coagulant dose is also important in removing MPs from water. As coagulant dose increased, all coagulant types exhibited an initially increasing and then stabilizing trend in MP removal (Ma et al., 2019a; Ma et al., 2019b; Lapointe et al., 2020; Shahi et al., 2020; Xue et al., 2021; Zhou et al., 2021). Charge neutralization and sweep flocculation are the two possible mechanisms of particle removal in CFS. Given the applied coagulant doses and the relatively large sizes of MPs in these studies (e.g., Ma et al. (2019b) and Zhou et al. (2021) used MPs at mm-scale), the MP removal in these studies was likely through sweep flocculation (Ma et al., 2019b; Xue et al., 2021). Thus, higher coagulant doses are beneficial to stronger sweep flocculation and better MP removal. When the coagulant dose reaches a certain level, the removal of MPs may, however, be reduced (Shahi et al., 2020; Zhou et al., 2021).

**Table 3** Laboratory-scale studies on MPs in DWT or surface water treatment

| Study | Country | Source water | MPs | Dispersant/ surfactant | Treatment process | Coagulant and aid | Coagulant aid | Membrane | Best removal (%)* |
|---|---|---|---|---|---|---|---|---|---|
| Zhou et al. (2021) | China | Synthetic water | Crushed PS (1.05 g/cm$^3$) and PE (0.91 g/cm$^3$) size: < 5 mm | – | CFS | PACl (30–180 mg/L) | | | PS: ~80% when PAC$\geqslant$60 mg/L PE: ~30% when PAC$\geqslant$90 mg/L |
| | | | | | | $FeCl_3$ (30–180 mg/L) | | | PS: ~65% when $FeCl_3\geqslant$60 mg/L PE: ~16% when $FeCl_3\geqslant$90 mg/L |
| | | | | | | ACH: 0–3.85 mg/L | | | Similar removal of microplastics as compared with alum |
| Skaf et al. (2020) | USA | Synthetic water | Model PE microspheres density: 1.3 g/cm$^3$ diameter: 1–5 µm surface: pristine | F68 | CFS | Alum: 5–10 mg Al/L | | | – |
| | | | Model PE fibers density: 0.96 g/cm$^3$ diameter: 5 µm (0.1 mm long), 15 µm (0.9 and 1.3 mm long) surface treatment: polyvinyl alcohol | F68, Alconoc, Tide Oxi Ultra, or All Stainlifter | | | | | – |
| Xia et al. (2020) | China | Synthetic water | PS microspheres size: 1, 2, 3, 4, and 5 µm | Tween 20 or sodium dodecyl sulfate (SDS) | CFS | $AlCl_3\cdot6H_2O$, 0–0.5 g/L | | | No surfactant: ~98% removal of 1 µm MP at 0.25 g/L $AlCl_3\cdot6H_2O$; Tween led to reduced removal of 1 µm microspheres in contrast to SDS |
| Xue et al. (2021) | Canada | River water | Carboxylated PS microspheres size: 3, 6, 25, 45, and 90 µm; density: 1.05 g/cm$^3$ | - | CFS | Alum: 0–50 mg/L | | | 50 mg/L alum: 3 µm: 95.7% 6 µm: 91.2% 25 µm: 97.7% 45 µm: 89.9% 90 µm: 80.5% |
| | | River water | | | | Alum: 30 mg/L | | | 3 µm: 85.2% 6 µm: 75.6% 25 µm: 77.2% 45 µm: 63.3% 90 µm: 44.5% |
| | | Prechlorinated lake water | | | | Alum: 30 mg/L | | | 3 µm: 96.1% 6 µm: 85.2% 25 µm: 90.6% 45 µm: 88.0% 90 µm: 63.7% |
| Lapointe et al. (2020) | Canada | River water | PE microspheres: 15 and 140 µm Weathered PE microspheres: 64 µm PS microspheres: 140 µm PEST fibers: $\leqslant$63 µm in length and 90 µm in diameter | – | CF-ballasted flocculation | Alum: 0–3.85 mg Al/L | | | PE (140 µm): ~90% PE (15 µm): ~92% Weathered PE (64 µm): ~97% PS (140 µm): 83.7% (2.71 mg Al/L) PEST fiber: 100% |

(*Continued*)

| Study | Country | Source water | MPs | Dispersant/ surfactant | Treatment process | Coagulant and aid | Coagulant aid | Membrane | Best removal (%)* |
|---|---|---|---|---|---|---|---|---|---|
| Ma et al. (2019b) | China | Synthetic water | Model PE microspheres, density: 0.92–0.97 g/cm$^3$, diameter: < 0.5–5 mm | Humic acid (HA) | CFS | $FeCl_3 \cdot 6H_2O$: 0–5 mM | | | 2 mM $FeCl_3 \cdot 6H_2O$ at pH 7.0: d < 0.5 mm: 13.5% 0.5 < d < 1 mm: 6.4% 1 < d < 2 mm: 3.7% 2 < d < 5 mm: 2.4% |
| | | | | | CFS | $AlCl_3 \cdot 6H_2O$: 0–15 mM | | | 15 mM $AlCl_3 \cdot 6H_2O$ at pH 7.0: d < 0.5 mm: 36.5% 0.5 < d < 1 mm: 20.6% 1 < d < 2 mm: 11.5% 2 < d < 5 mm: 4.4% |
| | | | | | CFS | $AlCl_3 \cdot 6H_2O$: 0.5 and 5 mM | cationic PAM: 0–15 mg/L | | 5 mM $AlCl_3 \cdot 6H_2O$ and 15 mM cationic PAM at pH 7.0: d < 0.5 mm: 45.7% 0.5 < d < 1 mm: 21.3% 1 < d < 2 mm: 9.3% 2 < d < 5 mm: 5.7% |
| | | | | | CFS | $AlCl_3 \cdot 6H_2O$: 0.5 and 5 mM | anionic PAM: 0–15 mg/L | | 5 mM $AlCl_3 \cdot 6H_2O$ and 15 mM anionic PAM at pH 7.0: d < 0.5 mm: 61.3% 0.5 < d < 1 mm: 41.3% 1 < d < 2 mm: 30.0% 2 < d < 5 mm: 17.7% |
| | | | | | CFS-UF | $FeCl_3 \cdot 6H_2O$ | | PVDF 100 kDa UF | – |
| | | | | | UF | | | PVDF 100 kDa UF | – |
| Ma et al. (2019a) | China | Synthetic water | Model PE microspheres, density: 0.92–0.97 g/cm$^3$, diameter: < 0.5–5 mm | HA | CFS | $FeCl_3 \cdot 6H_2O$: 0–5 mM | | | 2 mM $FeCl_3 \cdot 6H_2O$ at pH 7.0 d < 0.5 mm: 13.2% 0.5 < d < 1 mm: 6.5% 1 < d < 2 mm: 3.8% 2 < d < 5 mm: 2.3% |
| | | | | | CFS | $FeCl_3 \cdot 6H_2O$: 0.2 and 2 mM | cationic PAM: 0–15 mg/L | | 2 mM $FeCl_3 \cdot 6H_2O$ and 15 mg/L cationic PAM at pH 7.0 d < 0.5 mm: 55.9% 0.5 < d < 1 mm: 27.3% 1 < d < 2 mm: 15.9% 2 < d < 5 mm: 5.7% |
| | | | | | CFS | $FeCl_3 \cdot 6H_2O$: 0.2 and 2 mM | anionic PAM: 0–15 mg/L | | 2 mM $FeCl_3 \cdot 6H_2O$ and 15 mg/L anionic PAM at pH 7.0 d < 0.5 mm: 88.6% 0.5 < d < 1 mm: 86.9% 1 < d < 2 mm: 86.9% 2 < d < 5 mm: 83.7% |
| | | | | | CFS-UF | $FeCl_3 \cdot 6H_2O$: 0.2and 2 mM | | PVDF 100 kDa UF | – |
| | | | | | UF | | | PVDF 100 kDa UF | – |

(*Continued*)

| Study | Country | Source water | MPs | Dispersant/ surfactant | Treatment process | Coagulant and aid | Coagulant aid | Membrane | Best removal (%)* |
|---|---|---|---|---|---|---|---|---|---|
| Shahi et al. (2020) | Korea | Synthetic water | PE particles, 10–100 μm 0.91 g/cm$^3$ | HA | CFS with cationic polyamine coated sand | Alum: 10, 20, 30, 40, and 50 mg/L | Cationic polyamine: 0.5, 1, and 2 mg/L | | ~71% removal of total MPs at 30 mg alum/L; For smaller MPs, 10–30 μm, the maximum removal 52% was at 30 mg alum/L |
| Lu et al. (2021) | China | Synthetic water | Pristine and weathered PET microspheres: Diameter: 500±2.5 nm | – | CFS | PACl ($Al_{13}$) | – | – | Pristine PET: 100% (by mass concentration) at pH = 6; Weather PET: 90% (by mass concentration) at pH = 8 |
| Park et al. (2021) | Korea | Synthetic water | Chitosan and tannic acid pre-coated MPs: PS: 0.5 μm and 90 μm; PE: 45–53 μm and 106–125 μm | – | Coagula-tion-mem-brane filtra-tion | $FeCl_3$ | – | 11-μm filter paper | 0.5-μm PS beads: 97% (by mass concentration) |
| | | | | | CFS | $FeCl_3$ | – | – | 0.5-μm PS beads: 50%–60% (by mass concen-tration) |
| | | | | | CFS | $AlCl_3$ | – | – | 0.5-μm PS beads: 45%–57% (by mass concen-tration) |
| Peydayesh et al. (2021) | Switzer-land | Synthetic water | Carboxylated PS microspheres Diameter: 500 nm | – | CFS | Lysozyme amyloid fibrils | – | – | 98.2% (by turbidity) at 12.5 mg/L coagulant |
| | | Primary effluent from a WWTP | Carboxylated PS microspheres: Diameter: 500 nm | – | CFS | Lysozyme amyloid fibrils | – | – | 81% (by turbidity) at 300 mg/L coagulant |
| Zhang et al. (2021b) | China | Synthetic water | Crushed PET Size: < 100–500 μm | – | CFS | PACl: 20–200 mg/L | PAM: 0–100 mg/L | – | 79.35% at 20 mg PACl/L and 100 mg PAM/L |
| | | | | | CFS | PACl: 20–200 mg/L | Sodium alginate (SA): 0–100 mg/L | – | 69.9% at 20 mg PACl/L and 100 mg SA/L |
| | | | | | CFS | PACl: 20–200 mg/L | Activated silicic acid (ASA): 0–100 mg/L | – | 69.8% at 20 mg PACl/L and 100 mg ASA/L |
| Li et al. (2021) | Singapore | Tap water or raw water | PS microspheres, 0.1, 1, 10, 18 μm; 1.05 g/cm$^3$ | | UF | | | PVDF hollow fiber membrane, 0.03 μm pore size | |
| | | | | | Coagula-tion-UF | $AlCl_3 \cdot 6H_2O$ | | PVDF hollow fiber membrane, 0.03 μm pore size | |
| Wang et al. (2020a) | UK | Synthetic water | PS microspheres: 10 μm | – | Biochar filter | | | | > 95% removal |
| | | | | | Sand filter | | | | 60%–80% removal |
| Li et al. (2020) | China | Synthetic water | PVC, < 5 μm | | MBR | | | 0.1 μm pore size | Almost complete removal of PVC MPs |
| Enfrin et al. (2020) | UK | Synthetic water | PE MPs and NPs from personal facial scrub product Size: 13–690 nm | – | UF | – | – | Polysulfone 30kDa | 54% (by nanoparticle tracking analysis) at operation time of 24 h |

The decline in MP removal at higher coagulant doses may be due to the looser and more fragile structures of larger flocs. To better understand the interactions between MPs and coagulant flocs in CFS, Zhou et al. (2021) conducted a number of analyses, such as surface charge, scanning electron microscopy (SEM), and FTIR, and gained some interesting findings such as the effects of electrostatic interaction, physical and chemical adsorption between MPs and flocs. Such efforts using real or more environment relevant MPs are bound to reveal more insights.

To enhance the CF treatment of MP-laden water, various coagulant aids, such as polyacrylamide (PAM), have been used in DWT. Ma et al. (2019a); Ma et al. (2019b), and Shahi et al. (2020) used coagulant aids in their CFS experiments for MP removal. Ma et al. (2019a) and Ma et al. (2019b) found that the addition of PAM (3–15 mM) influenced minimally the removal of PE MPs when the coagulant doses were low (such as 0.2 mM $FeCl_3 \cdot 6H_2O$); however, the PE removal was significantly improved by PAM addition when the coagulant dose was higher (e.g., 2 mM $FeCl_3 \cdot 6H_2O$). In addition, these two studies both indicated that anionic PAM was more beneficial to the removal of PE MPs than cationic PAM, which was attributed to the stronger electrostatic interaction between the positively charged Al-flocs at pH 7.0 and anionic PAM. It should be noted that the PAM doses in Ma et al. (2019a) and Ma et al. (2019b) were above the allowed level of 1.0 mg/L set by the World Health Organization (WHO) (World Health Organization, 2003). In Shahi et al. (2020), the authors used cationic polyamine coated sand (PC-sand) to enhance alum-based CFS treatment for PE MP removal, and found that the use of PC-sand improved the removal of PE MPs by 27%. Hence, the use of coagulant aid can improve the removal of MPs. Systematic investigation regarding a number of questions surrounding this remains lacking, such as to what extent coagulant aids are influencing MP removal and how the coagulant type, dose, other treatment conditions, and MPs themselves co-determine the enhancement of MP removal by using coagulant aids.

### 3.2 Effects of MP material, size, shape, and surface properties on MP removal

The removal of particles in CFS is largely related to their settleability or affinity to the flocs formed in coagulation and flocculation, so the morphological, physical, and chemical properties of MPs are important in determining their removal in CFS. Intuitively, the MP materials, particle size, shape, and surface properties are influential factors to the removal of MPs in CFS.

However, the polymer type of MPs may be less likely an influential factor to MP removal in CFS. Unlike bare polymer particles used in most laboratory MP studies, MPs in natural water are often weathered or associated with other materials, such as NOM and biofilms. Thus, the density, surface morphology, and surface chemical properties can be very different from their pristine polymers (Lapointe et al., 2020). Because of this, MPs with similar shapes and sizes rather than chemical origins may end up being very similar in terms of their behavior in CFS. Zhou et al. (2021) attributed the better removal of model PS MPs than their PE counterparts to the higher density of PS. Lapointe et al. (2020) observed similar removals of 140-μm PE and PS microspheres at 2.73 mg Al/L (alum). The authors concluded that the removal of MPs is more influenced by weathering than by the polymer types. To date, full-scale studies usually compare the relative abundance of different polymers in the raw and treated water, but rarely compare the removal of MPs of different polymers (Pivokonsky et al., 2018; Pivokonský et al., 2020). Wang et al. (2020a) is perhaps the only study so far that has explored the impact of polymer types on MP removal at a full-scale DWTP. They found the MP removals were 87.4 (PET), 9.1 (PE), 14.3 (PP), and 16.9% (others) by the entire DWTP, with the PET MPs being mostly fibrous. However, the authors did not compare the removals of MPs of different materials specifically in CFS. Although the materials of MPs may determine their mechanical and chemical stability, they may not be the direct influencers to the removal of MPs in CFS.

Shapes of MPs seem to influence their removal in CFS. Fibrous MPs are generally more effectively removed by CFS. In Wang et al. (2020a), fibers were the most effectively removed MPs in CFS (~50.7%–60.6%). Shahi et al. (2020) found that MP fibers were better removed than spheres. Lapointe et al. (2020) reported constantly better removal of PEST fibers than PE and PS spheres from river water by CFS over a range of alum and PAM doses. In Pivokonský et al. (2020), 59.5% of fibrous MPs were removed by CFS. The better removal of fibrous MPs in CFS may be explained by: 1) fibrous particles are more likely to form flocs and settle (Katrivesis et al., 2019); 2) fibrous MPs are usually heavier polymers, such as nylon (density 1.15 $g/cm^3$), PEST (density 1.38 $g/cm^3$), CA (density 1.30 $g/cm^3$), or PET (density 1.38 $g/cm^3$); and 3) the polymers consisting fibrous MPs often contain chemical groups (such as C = O of PEST and PET) that facilitate their attachment to coagulant flocs. In addition, surface roughness of MPs is also influential to their removal with the rougher being better removed (Shahi et al., 2020).

Size also matters to the removal of MPs by CFS. The effect of particle size on MP removal has been discussed in most of the published studies, and seemingly contradictory findings are provided. Shahi et al. (2020) found that model PE MPs within 10–30 μm were remarkedly harder to remove than larger ones (30–100 μm) from synthetic water. In contrast, Xue et al. (2021)'s laboratory-scale alum-based CFS experiments using real surface waters and model PS microspheres (3–90 μm) with carboxylated surfaces indicated better removal of smaller MPs than the larger ones. Lapointe et al. (2020) also observed poorer removal of larger PE MPs (140 μm) than the smaller (15 μm). Prior to Xue et al. (2021) and Ma et al. (2019a); Lapointe et al. (2020) and Ma et al. (2019b) found that larger PE MPs were more effectively removed from synthetic water by CFS; however, the MP sizes used in the studies were relatively large (0.5–5 mm) and less representative of the dominant MP sizes in real DWTP raw water. For larger MPs (such as ~100 μm), larger flocs are required to capture them for effective removal (Lapointe et al., 2020). As sweep flocculation was the dominant mechanism in these aforementioned laboratory studies, the discrepancies regarding the effect of MP size on their removal by CFS were likely caused by the difference in water characteristics, model MP properties (pristine vs. weathered or carboxylated surfaces), and CFS operating conditions. Although the full-scale studies also discussed the effect of particle size on MP removal by CFS, it is difficult to draw clear solutions due to the compounded properties of MPs in natural waters. For example, in Wang et al. (2020a), MPs>10 μm were better removed than those within 5–10 μm, but the authors also clarified that the majority of MPs>10 μm were fibers and those within 5–10 μm were largely fragments. To reveal the effect of MP size on their removal in CFS treatment, more investigations are needed under controlled conditions.

### 3.3 Effect of water quality on MP removal

Water quality is influential to the removal of MPs by CFS. pH, NOM, ionic strength, divalent cations, and particulate/colloidal matter of source waters can differ greatly in different locations and seasons. Complex physical, chemical, and biological processes may remarkably alter the surface morphology and properties of MPs. For instance, NOM and divalent cations may be adsorbed on weathered MP surfaces through hydrophobic interaction, electrostatic interaction, or complexation (bridging). Thus, source water characteristics conceivably affect the association of MPs and coagulant flocs. To date, very few studies have looked into the effect of water quality on MP removal by CFS. Xue et al. (2021) compared two distinct surface waters for MP removal; they found that higher turbidity favored the formation of larger and denser flocs and resultantly better removal of MPs. In contrast, Ma et al. (2019b) found no apparent influence of water characteristics on the removal of PE MPs. It should be noted that this study used simple synthetic water and un-weathered model PE particles (0.5–5 mm), which was not representative of the treatment of real water. So far, it remains largely unclear in detail how water quality impacts the removal of MPs with different characteristics by CFS.

### 3.4 Effects of MPs on other parameters in CFS treatment

To date, two studies have examined the impacts of MPs on CFS treatment (Lapointe et al., 2020; Xue et al., 2021). Although both studies used MP concentrations that were higher than environmental levels, specifically, 500 MPs/L in Lapointe et al. (2020) and ~60 – ~239 MPs/L in Xue et al. (2021), MPs did not impact the aggregation mechanisms or coagulant demand, which disagrees to the hypothesis established by Enfrin et al. (2019). Xue et al. (2021) dosed PS MPs with carboxylated surfaces and a size range of 3–90 μm in real river water and lake water and investigated the impacts of MPs on the CFS treatment in terms of turbidity reduction, pH change, removal of NOM fractions, and major metal elements (such as Ca, Mg, and Cu). No perceivable impact of MPs has been observed on any of these aspects of CFS treatment performance. Therefore, given the usually low concentrations of MPs in natural water, MPs may be less likely an influencer to CFS treatment. Nevertheless, the impact of MPs on trace organic pollutants in CFS remains to be studied due to the potentially strong interaction between them. Recently, Lu et al. (2021) investigated the interaction between PET MPs and tetracycline in the coagulation process and found that the removal of PET was significantly decreased by the presence of tetracycline. Therefore, the potential interactions between MPs and trace pollutants are worth thorough investigation.

## 4 MP removal by other processes

After CF (or CFS), there are usually some additional treatment processes at DWTPs, such as membrane filtration, granular filtration, and disinfecting. Since CF cannot completely remove MPs from water, the subsequent treatment processes are worth investigation in their efficiency of removing MPs. There are a few other studies that analyzed the removal of MPs by membrane filtration and media filtration (Table 1 and Table 3). This section will discuss the removal and impact of MPs in these treatment processes.

### 4.1 Membrane filtration

Membrane technology has been widely used in DWT, which usually comes after the CF or CFS treatment. So far, less than 10 studies have explored the removal and impacts of MPs in membrane processes in laboratory-scale DWT (Ma et al., 2019a; Ma et al., 2019b; Li et al., 2020; Li et al., 2021). Ultrafiltration (UF), microfiltration (MF), or reverse osmosis (RO) membranes have been studied in these studies. Even fewer studies report MP removal by membranes at full-scale DWTPs (Johnson et al., 2020;

Dalmau-Soler et al., 2021). Since the studied MPs were mostly larger than the membrane pore sizes (Table 3), complete removal of MPs was reported in almost all of these studies. In Dalmau-Soler et al. (2021), the DWTP contains UF + RO in parallel with ozonation + GAC filtration before combining the two lines for chlorination; however, the researchers did not specifically measure MPs in the UF or RO effluent. Hence, no detailed information of MP removal by UF or RO can be drawn from that study. It remains unknown how smaller MPs ( < membrane pore sizes) and MPs with different shapes (such as fibers), behave in various membrane filtration processes (particularly low-pressure driver membranes) in DWT.

In membrane filtration, a number of mechanisms, such as size exclusion, hydrophobic interactions, and electrostatic repulsion forces, are involved in removing particles from water. Thus, the characteristics of MPs (such as size, shape, and polymer type) and membranes (such as structure, pore size, and membrane material) can affect the removal of MPs. In Cai et al. (2020a), membrane filters of different materials, structures, and pore sizes demonstrated distinct retaining capabilities for MPs. In addition, the authors found that large fibers (3568 μm) remained after filtration with a membrane filter with a pore-size of 1000 μm, whereas 37.2-μm MP fragments were observed on membrane filters with 50-μm pores. It appears that fibrous MPs are more likely to pass through membranes when the smaller dimensions of the fibers are smaller than the membrane pore size. In Michielssen et al. (2016), fibers accounted for ~80% of the remaining MPs in the effluent from a tertiary wastewater treatment consisting of sand filtration and MBR, in contrast to 44% in the effluent of activated sludge process. To date, research on the fate and transport of MPs of different characteristics in membrane processes, especially low-pressure membranes, under various DWT conditions, is largely lacking.

Membrane fouling is the major challenge for membrane technology. Previous research indicated that MPs could influence membrane fouling. Without CFS pretreatment, the MPs can increase membrane fouling with the smaller MPs or higher MP concentrations generally leading to severer membrane fouling (Ma et al., 2019a; Ma et al., 2019b; Li et al., 2021). On the other hand, when CFS pretreatment was in place, Ma et al. (2019a) and Ma et al. (2019b) found that the PE MPs alleviated the fouling caused by the coagulant flocs, with the larger MPs being more beneficial. As for the effect on membrane fouling of different MP sizes, Li et al. (2021) documented that the 1-μm MPs led to the worst membrane fouling among the 0.1-, 1-, 10-, and 18-μm MPs, suggesting a critical MP size of 1 μm for their membrane filtration. Moreover, MPs can enhance organic fouling and worsen the irreversibility of fouling according to Li et al. (2020). By examining the UF membrane fouling induced by NPs (13–690 nm), Enfrin et al. (2020) found that 38% reduction in permeate water flux was caused by the NPs over 48 h at 1-bar, and hydrophobic interactions and surface repulsion forces dictated the adsorption process of the NPs onto the polysulfone UF membrane surface. It should be noted that to date the studies on MPs in membrane process were mostly based on simple water matrices and model MPs with unified shapes, pristine surfaces, and micrometer-scale sizes. The behavior and impact on membrane fouling of MPs with different shapes, weathered surfaces, associated pollutants, and smaller sizes remain to be explored under various membrane filtration conditions. Moreover, most membranes used at DWTPs are polymeric, making them a potential MP source in drinking water (Dalmau-Soler et al., 2021; Ding et al., 2021). The contribution of polymeric membranes to MPs in treated water ought to be assessed.

### 4.2 Media filtration

Media filtration can effectively remove MPs. The commonly employed media filtration at DWTPs are sand filtration and GAC filtration. According to Wang et al. (2020a), sand filtration removed 31%–49% of MP fibers, 24%–51% of MP spheres, and 19%–28% of MP fragments from the sedimentation effluent, which were lower than the preceding CFS. However, MPs > 10 μm in the CFS effluent could be completely removed by sand filtration. Thus, the poor removal of larger MPs by CFS reported in Xue et al. (2021) (>45 μm) and Lapointe et al. (2020) (140 μm) should not be a concern at full-scale DWTPs. On the other hand, the residual small MPs (e.g., 5–10 μm) after CFS can be a challenge to sand filtration (Pivokonský et al., 2020). The persistence of small MPs through sand filtration suggests the necessity of enhancing CFS treatment or implementing additional treatment for removing smaller MPs. GAC filtration has been used at some advanced DWTPs as a final polishing step, which proved effective in removing MPs with different sizes and shapes (Pivokonsky et al., 2018; Pivokonský et al., 2020; Wang et al., 2020a). Wang et al. (2020a) found that the GAC filtration could remove 57%–61% of the residual MPs in the water, 74%–99% of the removed MPs were within the size range of 1–5 μm, and fibrous MPs were the least removed by GAC among all shapes (45% fibers vs. 82% of spheres). According to granular media filtration theories, particle removal in media filtration involves transport, attachment, and detachment (Emelko et al., 2005). Through transport mechanisms, mainly diffusion and sedimentation, particles are brought close enough to collectors (i.e., filter media that may or may not have previously deposited particles) so that attachment can happen (O'Melia and Stumm, 1967). Resultantly, particles with a size near 1 μm are the most persistent through media filtration due to the minimum net transport efficiency (Amirtharajah, 1988). Due to the lack of research, it remains unknown how different media filtration conditions, such as media size and surface properties, filtration rate, temperature, and backwashing cycles, impact the removal of MPs (with different sizes, shapes, and surface morphologies and properties) in different water matrices (e.g., NOM, colloids, and ionic strengths). It is also interesting to investigate how MPs with distinct properties behave in media filtration in terms of their respective ripening, effective filtration, breakthrough, and wormhole flow stages. Some insights can be drawn from previous studies on the removal of protozoa using surrogate microspheres (Tufenkji et al., 2004; Brown and Emelko, 2009; Gottinger et al., 2013). For instance, a study on the removal of microspheres surrogating water-borne protozoa in media filtration showed that higher flow rates enhanced the transport of MPs in media filters, and NOM adsorption on media grains promoted the mobility of MPs through the filters (Zhang et al., 2017). Yet, these studies used microspheres with a size of 3–6 μm to mimic *Cryptosporidium* oocysts, which is less representative of the more diverse MPs in DWT. Therefore, for better removal of MPs through granular media filtration, more research is due.

In addition to membrane filtration, sand filtration, and GAC filtration, the impact of ozonation on MP removal has also been revealed by some studies (Pivokonsky et al., 2018; Pivokonský et al., 2020; Wang et al., 2020a; Dalmau-Soler et al., 2021). Ozonation's influence on MP concentration is minimal (Pivokonsky et al., 2018; Pivokonský et al., 2020). Wang et al. (2020a) found slightly increased MP concentration after ozonation, which could be attributed to the breakdown of larger MPs and destruction of the residual organic matter covering MPs (thus better detectability). Nonetheless, the size, shape, and surface properties of MPs are anticipated to be changed by ozonation, which may influence the removal of MPs in subsequent treatment processes.

## 5 Conclusions and recommendations

Although there are very limited studies published on MPs in DWT, the preliminary findings have indicated that the typical DWT trains, including the basic media filtration series, CFS-sand filtration, and CFS-sand filtration-ozonation-GAC filtration, are effective in reducing the concentration of MPs in drinking water. Because of the high diversity of MPs in water, DWTPs with more steps are more effective than simpler treatment trains (such as CF-sand filtration) in terms of MP removal. The key findings in this literature research are as follows.

1) MPs in drinking water source water are very diverse in terms of concentrations, sizes, shapes, and materials. The commonly reported MP materials in drinking water source water are PE, PP, PS, PET, nylon, etc. Fragments, spheres, and fibers are the major shapes. The inconsistency in categorizing MP shapes makes it difficult to compare studies by different researchers. Thus, it is necessary to establish a set of standardized MP shape categories. As for size distribution, many studies were able to examine MPs with a size down to a few μm. The detection limit is the biggest challenge in MP studies. Without robust detection techniques, it is difficult to investigate plastic particles that are within the nanometer range, which could potentially be more persistent through DWTPs. With respect to MP concentrations, they can differ enormously between times and locations. Due to the high diversity and dynamics of MPs in each water body, each DWTP may need to establish a good understanding of the MP composition in their respective water source for more effective MP handling in practice.

2) As the step intended for particulate and colloidal pollutant removal, CFS is often the major contributor to MP removal in DWT. The shapes and sizes of MPs are influential to their removal by CFS. Fibrous MPs are generally easier to remove by CFS than spherical and fragmental MPs, which is related to their higher density and stronger tendency of forming flocs. As for sizes, within the range of 1–100 μm, it seems that smaller MPs are more effectively removed. In addition, the characteristics of water can impact the removal of MPs as the other substances in water, such as NOM and divalent cations can interact with MPs and change their behavior in the coagulation-flocculation processes. Moreover, coagulant type and dose, coagulant aid type and dose, and treatment conditions can be influential factors. The studies that are currently available are very preliminary and limited. More research is warranted to clarify how different MPs (size, shape, morphology, surface properties, and associated pollutants) are removed and influential in CFS under various conditions.

3) Membrane technology has been widely used in DWT globally. The preliminary studies showed that UF membranes can completely remove the examined MPs (>0.1 μm), which is anticipated as size exclusion is the major mechanism in membrane filtration. When the smaller dimensions of the fibrous MPs are smaller than the membrane pore size, fibers can be challenging to membrane filtration. As for the impact of MPs, they may contribute to membrane fouling with smaller MPs and higher concentrations being more influential. On the other hand, when CFS pretreatment is provided, MPs can alleviate membrane fouling caused by the coagulant flocs. To date, the removal of MPs by membrane filtration remains understudied. The removal and impact of MPs with different shapes, sizes, and surface properties remain to be studied. In addition, the release of MPs by membrane materials themselves should be evaluated.

4) Sand filtration and GAC filtration are the common media filtration processes in DWT. As a post-treatment to CFS, sand filtration can effectively remove the residual MPs in water, particularly the larger ones (such as >10 μm). However, it is less efficient in removing small MPs (such as < 10 μm). GAC filtration has been implemented at some DWTPs as an additional treatment process. It proved effective in removing residual MPs in water, with the smaller MPs (such as 1–5 μm) being the majority of the removed MPs. So far, the removal of MPs by sand filtration and GAC filtration is under-investigated. More efforts are needed to understand the removal and impacts of MPs with various properties in media filtration under various conditions.

To sum up, conventional and advanced DWTPs can remove the majority of MPs from water, but extensive research effort is still needed to understand and further enhance the removal of MPs in DWT processes. Despite the high diversity and variations of MPs in natural water, it is not necessary or practical to holistically examine all possible MP types. Based on the available studies, smaller MPs (such as < 10 μm), spherical and fragmented, are more challenging to conventional DWTPs. Therefore, future studies may need to focus on the behavior and impact of smaller spherical or fragmental MPs in various treatment processes under different conditions. In addition, fibrous MPs in membrane filtration ought to be further studied. Upon the availability of detection and quantification techniques for nanoplastics, their transport and fate in DWTPs should be explored.

**Acknowledgements** This study was supported by the Natural Sciences and Engineering Research Council of Canada (NSERC) through the NSERC Discovery Grants Program and NSERC Discovery Launch Supplement Grant. In addition, we would thank the support by the University of Regina through the New Faculty Start-Up Fund.

**Open Access** This article is licensed under a Creative Commons Attribution 4.0 International License, which permits use, sharing, adaptation, distribution and reproduction in any medium or format, as long as you give appropriate credit to the original author(s) and the source, provide a link to the Creative Commons licence, and indicate if changes were made. The images or other third party material in this article are included in the article's Creative Commons licence, unless indicated otherwise in a credit line to the material. If material is not included in the article's Creative Commons licence and your intended use is not permitted by statutory regulation or exceeds the permitted use, you will need to obtain permission directly from the copyright holder. To view a copy of this licence, visit http://creativecommons.org/licenses/by/4.0/.
